# Hemodynamic Effects of Controlled‐Release Nifedipine for Tocolysis in Normotensive Pregnant Women

**DOI:** 10.1111/jog.70560

**Published:** 2026-11-10

**Authors:** Takafumi Ushida, Sumire Fujito, Yoshitaka Shiraishi, Yuki Nozaki, Kazuya Fuma, Sho Tano, Seiko Matsuo, Hiroaki Kajiyama

**Affiliations:** ^1^ Department of Obstetrics and Gynecology Nagoya University Graduate School of Medicine Nagoya Japan; ^2^ Division of Reproduction and Perinatology, Center for Maternal‐Neonatal Care Nagoya University Hospital Nagoya Japan

**Keywords:** controlled‐release nifedipine, hypotension, maternal hemodynamics, threatened preterm labor, tocolysis

## Abstract

**Aim:**

Nifedipine is widely used as a tocolytic agent for threatened preterm labor. However, its hemodynamic effects in normotensive pregnant women, particularly when administered as a controlled‐release formulation, remain unclear. We evaluated changes in maternal hemodynamics and adverse effects after controlled‐release nifedipine administration in normotensive pregnant women with threatened preterm labor.

**Methods:**

This retrospective observational study included normotensive women with singleton pregnancies who received controlled‐release nifedipine as maintenance therapy for threatened preterm labor between 2018 and 2025. Maternal blood pressure, heart rate, respiratory rate, hypotension (systolic blood pressure < 90 mmHg or diastolic blood pressure < 60 mmHg), tachycardia (≥ 120 beats/min), and adverse maternal and fetal effects were evaluated before and after nifedipine administration.

**Results:**

Overall, 38 women were included in the final analyses. Systolic blood pressure significantly decreased from 110.2 ± 9.1 mmHg to 107.5 ± 10.2 mmHg after administration (mean change, −2.7 mmHg; 95% confidence interval, −4.5 to −0.8; *p* < 0.01). Diastolic blood pressure did not change significantly (−0.7 mmHg; −2.2 to 0.8 mmHg; *p* = 0.35). Heart rate significantly decreased from 95.3 ± 14.5 to 90.9 ± 12.4 beats/min. The incidence of hypotension and tachycardia did not differ significantly after nifedipine administration. No emergency deliveries were attributed to maternal hypotension. Headache was the most commonly reported adverse effect (28.9%).

**Conclusions:**

Controlled‐release nifedipine was associated with a small decrease in systolic blood pressure without serious maternal or fetal adverse events. With appropriate monitoring, controlled‐release nifedipine may be a feasible option for maintenance or adjunctive tocolytic therapy in normotensive pregnant women.

## 1 Introduction

Preterm birth is a leading cause of neonatal mortality and short‐ and long‐term neonatal morbidity, and preventing preterm birth remains a major challenge in perinatal medicine [1, 2]. Tocolytic therapy for threatened preterm labor is primarily intended to prolong short‐term pregnancy, thereby allowing time for maternal transfer and administration of antenatal corticosteroids to promote fetal lung maturation. Various tocolytic agents, including calcium channel blockers, beta‐adrenergic agonists, magnesium sulfate, oxytocin receptor antagonists, and indomethacin, have been used for this purpose [3]. Several international guidelines recommend nifedipine, a calcium channel blocker, as the first‐line or preferred agent for tocolysis [3, 4, 5, 6].

Nifedipine inhibits L‐type voltage‐dependent calcium channels, thereby reducing cellular calcium influx and suppressing uterine smooth muscle contractions. Compared with beta‐adrenergic agonists, nifedipine has fewer adverse maternal effects, comparable or superior tocolytic efficacy, and is widely used in clinical practice for threatened preterm labor [7, 8]. Nifedipine was originally developed as an antihypertensive agent; therefore, when administered to normotensive pregnant women, potential hemodynamic changes and adverse effects, such as hypotension, reflex tachycardia, headache, and palpitations, can occur. However, the changes in blood pressure after nifedipine administration for threatened preterm labor have not been sufficiently investigated in normotensive pregnant women.

Two major studies previously evaluated the hemodynamic effects of nifedipine as a tocolytic agent in normotensive pregnant women. Luewan et al. prospectively examined blood pressure changes up to 60 min after the administration of immediate release nifedipine in 157 normotensive pregnant women with threatened preterm labor [9]. Yamasato et al. also retrospectively evaluated changes in blood pressure and heart rate during the first 8 h of immediate release nifedipine administration in 138 nonhypertensive pregnant women receiving nifedipine for tocolysis [10]. These studies showed that mean systolic and diastolic blood pressures decreased modestly after nifedipine administration; however, no adverse fetal effects related to severe maternal hypotension were observed. These findings suggest that the observed hemodynamic changes were generally well‐tolerated in normotensive pregnant women.

However, the nifedipine formulations evaluated in previous studies were immediate release formulations, and these studies focused mainly on early hemodynamic changes during acute tocolysis, particularly from immediately after administration to 8 h after initiation [9, 10]. Controlled‐release nifedipine may have a more sustained tocolytic effect when used as a maintenance therapy during the initial 48 h after acute tocolysis. Controlled‐release formulations differ from immediate release formulations in their pharmacokinetic profiles, including the rate of increase and duration of plasma drug concentrations. Therefore, their effects on maternal blood pressure, heart rate, incidence of hypotension, and adverse effects may also differ. However, the hemodynamic effects of controlled‐release nifedipine in normotensive pregnant women have not been sufficiently evaluated.

Therefore, we aimed to clarify the hemodynamic effects of controlled‐release nifedipine in normotensive pregnant women and provide real‐world clinical data that may help guide its safe use and monitoring strategies in the management of threatened preterm labor.

## 2 Methods

### 2.1 Study Design and Participants

This retrospective observational study included normotensive pregnant women with singleton pregnancies between 22 + 0 and 36 + 6 weeks of gestation who received controlled‐release nifedipine as maintenance or adjunctive tocolytic therapy for threatened preterm labor at Nagoya University Hospital between January 2018 and December 2025. Women who were administered nifedipine in an outpatient setting and who had multiple pregnancies, hypertensive disorders of pregnancy, or were administered nifedipine at another hospital were excluded. In Japan, nifedipine has not been approved for the treatment of threatened preterm labor and is, therefore, used off‐label. Therefore, before administration, patients were informed that nifedipine was being used off‐label and the expected benefits and potential adverse effects were explained. Written informed consent for nifedipine use was obtained from all patients. The study protocol was approved by the Institutional Review Board of Nagoya University Hospital on March 4, 2016 (approval No. 2015‐0415). The present study was approved by subsequent protocol amendments, with the final approval granted on June 30, 2026. As this was a retrospective study with information obtained from medical records, the requirement for informed consent was addressed using an opt‐out approach.

### 2.2 Management Policy for Threatened Preterm Labor

Threatened preterm labor was defined as the presence of regular uterine contractions, cervical shortening, cervical dilatation, or a condition for which tocolytic therapy was prescribed by the attending physician. At our hospital, intravenous ritodrine hydrochloride is generally used as the first‐line tocolytic agent for threatened preterm labor. However, magnesium sulfate was considered in patients with contraindications to ritodrine hydrochloride, or in those for whom its use was considered inappropriate because of complications or adverse effects. Ritodrine hydrochloride and magnesium sulfate are used not only for acute tocolysis but also as maintenance therapy; however, dose reduction or discontinuation is considered when symptoms improve. Tocolytic agents are generally not used in patients with cervical shortening without uterine contractions, and management with vaginal progesterone is recommended. Controlled‐release nifedipine is mainly used as maintenance therapy or as an adjunctive therapy to other tocolytic agents. It has been used as an add‐on therapy in patients with insufficient control of uterine contractions despite existing tocolytic agents, as an alternative therapy in patients in whom continuation of the current tocolytic agent is inappropriate because of liver dysfunction or elevated creatine kinase levels, to reduce the dose of other tocolytic agents, or for supportive purposes when discontinuing intravenous tocolytic agents. Additionally, to characterize the pharmacokinetic profile of the controlled‐release nifedipine formulation used in this study, we summarized the pharmacokinetic parameters of controlled‐release nifedipine and compared them with those of immediate release and sustained release nifedipine formulations. The pharmacokinetic parameters after a single oral dose under fasting conditions in healthy adult men, including the maximum plasma concentration (*C*_max_), time to maximum plasma concentration (*T*_max_), elimination half‐life (*T*_1/2_), and area under the plasma concentration–time curve (AUC), are presented in Table S1 [11, 12, 13].

### 2.3 Nifedipine Administration and Discontinuation Criteria

The timing of initiation, dose, and duration of the controlled‐release nifedipine treatment were determined at the discretion of the attending physician based on the degree of uterine contractions, maternal blood pressure, heart rate, and the presence of adverse effects. Controlled‐release nifedipine was administered at 20‐mg doses, with the initial total daily dose ranging from 20 to 80 mg/day, at the discretion of the attending physician. The daily dose was adjusted according to the patient's clinical course. Blood pressure, heart rate, uterine contractions, and maternal symptoms were regularly assessed during treatment. If hypotension or adverse effects were suspected, temporary withholding, dose reduction, discontinuation, or switching to another tocolytic agent was considered. Discontinuation of controlled‐release nifedipine was determined by the attending physician based on the improvement in uterine contractions, clinical course of threatened preterm labor, gestational age, presence of adverse effects, and use of other tocolytic agents. However, the controlled‐release nifedipine was basically discontinued between 34 and 36 weeks of gestation.

### 2.4 Monitoring

After initiation of controlled‐release nifedipine, maternal blood pressure and heart rate were assessed regularly. The day of nifedipine administration was defined as Day 0. In the first 2 days after initiation (Days 0 and 1), blood pressure was measured before each dose in addition to routine vital sign measurements two to three times daily. From Day 2 onward, blood pressure was generally not measured before each dose; however, routine blood pressure measurements were taken two to three times daily. In the first 2 days after initiation, if the systolic blood pressure before administration was ≤ 90 mmHg, the scheduled dose of controlled‐release nifedipine was temporarily withheld. However, if the attending physician considers the administration necessary based on uterine contractions, and maternal and fetal conditions, nifedipine can be continued. From Day 2 onward, nifedipine was continued until obvious hypotension or other adverse effects were observed.

### 2.5 Outcomes

The primary outcomes were changes in maternal blood pressure and heart rate after initiation of controlled‐release nifedipine as well as the incidence of hypotension, severe blood pressure reduction, and tachycardia. Hypotension was defined as a systolic blood pressure < 90 mmHg or diastolic blood pressure < 60 mmHg. Severe hypotension was defined as a systolic blood pressure < 80 mmHg or diastolic blood pressure < 50 mmHg. For comparison with a previous study [9], severe blood pressure reduction was defined as a decrease of ≥ 15 mmHg from the baseline in either systolic or diastolic blood pressure. Tachycardia was defined as a heart rate ≥ 120 beats/min. The day of nifedipine initiation was defined as Day 0 and was excluded from the pre–post comparison. Changes in systolic blood pressure, diastolic blood pressure, and heart rate were evaluated by comparing the baseline period, defined as the week before nifedipine initiation (Days −7 to −1), with the postadministration period, defined as the week after initiation (Days 1 to 7). The baseline values were defined as the mean of all measurements obtained during the baseline period. If the baseline period before nifedipine initiation was < 7 days, all available measurements during that period were used to calculate baseline values. The post‐administration values were defined as the mean of all measurements obtained during the post‐administration period. For patients who delivered before Day 7, the data up to the day before delivery were analyzed. For patients who discontinued nifedipine before Day 7, data up to 24 h after nifedipine discontinuation were analyzed.

Secondary outcomes were changes in respiratory rate, peripheral oxygen saturation (SpO_2_), body temperature, incidence of adverse maternal effects, temporary withholding or discontinuation of nifedipine due to hypotension or adverse effects, and emergency delivery related to maternal hypotension. Respiratory rate, SpO_2_, and body temperature were evaluated in the same manner as blood pressure and heart rate, using the mean of all measurements recorded during each observation period. Maternal adverse effects, including headaches, palpitations, tachycardia, facial flushing, dizziness, nausea, fatigue, hypotension symptoms, and other adverse events documented by physicians or nurses, were collected from medical records. Cardiotocography and medical records were retrospectively reviewed to assess emergency cesarean delivery for fetal heart rate abnormalities potentially associated with maternal hypotension after nifedipine administration and the need for intrauterine resuscitation.

### 2.6 Sub‐Analysis of Maternal Hemodynamics Around the Expected Peak Plasma Concentration

To evaluate maternal hemodynamic changes around the expected peak plasma concentration after the administration of controlled‐release nifedipine, we conducted an additional sub‐analysis. Because the Tmax of 20 mg controlled‐release nifedipine is 3.1 ± 2.6 h, we focused on the time window expected to encompass the peak plasma concentration after administration. Among these 38 patients, those who received controlled‐release nifedipine after breakfast were also identified. Assuming a postbreakfast administration time of 8:30 AM, we included patients whose vital signs were measured between 9:30 AM and 2:00 PM after treatment initiation. This yielded 111 measurements in 28 patients. For comparison, we extracted the vital sign measurements obtained during the same time window (9:30 AM to 2:00 PM) in the same 28 patients before the initiation of controlled‐release nifedipine, yielding a total of 120 measurements. Using these pre‐ and posttreatment measurements, we compared maternal hemodynamic parameters during the time window corresponding to the expected peak plasma concentration.

### 2.7 Statistical Analysis

Continuous variables for baseline and clinical characteristics are presented as medians with interquartile ranges (IQR). Categorical variables are presented as numbers and percentages. Changes in maternal vital signs were evaluated before and after nifedipine administration. Systolic blood pressure, diastolic blood pressure, heart rate, respiratory rate, SpO_2_, and body temperature before and after administration are presented as mean ± standard deviation. Changes in each parameter were calculated as post‐administration values minus preadministration values and presented as mean changes with 95% confidence intervals (CI). For continuous variables, multiple measurements obtained during each observation period were averaged for each patient, and the resulting patient‐level mean values before and after nifedipine administration were compared using paired *t*‐tests.

For hypotension and tachycardia, the number and percentage of patients with at least one measurement meeting each criterion during the pre‐administration and post‐administration observation periods were calculated. The criteria were used to evaluate systolic blood pressure < 90 mmHg, systolic blood pressure < 80 mmHg, diastolic blood pressure < 60 mmHg, diastolic blood pressure < 50 mmHg, and heart rate ≥ 120 beats/min. Categorical variables were compared before and after administration using McNemar's exact test. Statistical significance was set at *p* < 0.05. Statistical analyses were performed using SPSS software version 29 (IBM Corp., Armonk, New York, USA).

### 2.8 Language Proofreading

ChatGPT (OpenAI, GPT‐5.6) was used exclusively for English‐language proofreading.

## 3 Results

During the study period, 54 pregnant women received controlled‐release nifedipine for threatened preterm labor. After excluding 16 women, 38 patients were included in the final analysis (Figure 1). The baseline characteristics of the study population are shown in Table 1. The median maternal age was 32.0 years (IQR, 28.3–35.0 years), and the median gestational age at nifedipine initiation was 31.0 weeks (IQR, 27.6–32.5 weeks). The median duration of nifedipine treatment was 24.5 days (IQR, 6.5–39.0 days). The median number of measurements per patient and the total number of measurements for each parameter before and after nifedipine administration are shown in Table S2.

**FIGURE 1:**
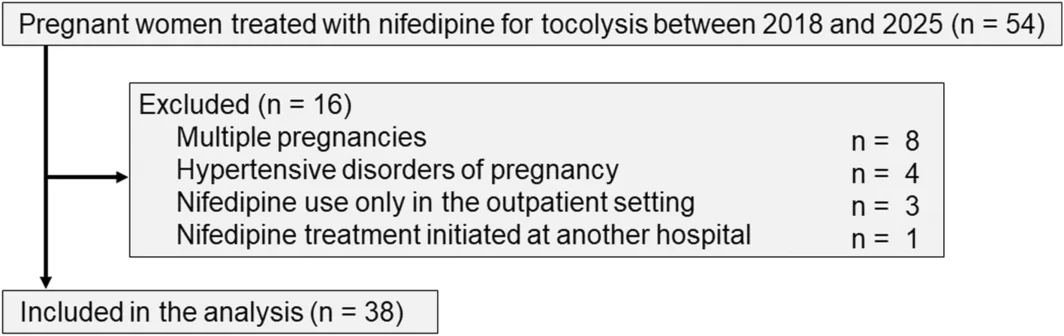
Flow diagram of the study population. We identified pregnant women who received controlled‐release nifedipine as a tocolytic agent between 2018 and 2025. After excluding women with multiple pregnancies, hypertensive disorders of pregnancy, nifedipine use exclusively in outpatient settings, or nifedipine treatment initiated at another hospital, 38 women were included in the final analysis.

**TABLE 1:** Maternal and neonatal characteristics of normotensive pregnant women treated with controlled‐release nifedipine for threatened preterm labor.

Maternal characteristics	
Maternal age (years)	32.0 (28.3 to 35.0)
Nulliparity	25 (65.8)
Prepregnancy body mass index (kg/m^2^)	20.2 (18.3 to 22.6)
Indications for nifedipine use	
Uncontrolled uterine contractions despite other tocolytic agents	7 (18.4)
Worsening adverse effects of other tocolytic agents	3 (7.9)
Liver dysfunction or elevated creatine kinase	5 (13.2)
To reduce the dose of other tocolytic agents	5 (13.2)
To discontinue other tocolytic agents	16 (42.1)
Other reasons	2 (5.3)
Gestational age at nifedipine initiation (weeks)	31.0 (27.6 to 32.5)
Gestational age at nifedipine discontinuation (weeks)	35.2 (31.7 to 36.0)
Duration of nifedipine treatment (days)	24.5 (6.5 to 39.0)
Daily dose of nifedipine at treatment initiation	
20 mg	16 (42.1)
40 mg	13 (34.2)
60 mg	2 (5.3)
80 mg	7 (18.4)
Maximum daily dose of nifedipine	
20 mg	15 (39.5)
40 mg	11 (28.9)
60 mg	5 (13.2)
80 mg	7 (18.4)
Reasons for nifedipine discontinuation	
Improvement of uterine contractions	2 (5.3)
Resumption of other tocolytic agents	6 (15.8)
Reaching the target gestational age	17 (44.7)
Delivery	13 (34.2)
Hypotension or adverse effects	0 (0)
Concomitant intravenous ritodrine	21 (55.3)
Concomitant intravenous magnesium sulfate	9 (23.7)
Gestational age at delivery (weeks)	36.7 (32.5 to 37.6)
Cesarean delivery	19 (50.0)
Prenatally suspected fetal abnormalities	12 (31.6)
Neonatal characteristics	
Male	19 (50.0)
Birth weight (g)	2384 (1829 to 2955)

*Note:* Data are presented as the median (interquartile range) or *n* (%). Prenatally suspected fetal abnormalities included congenital diaphragmatic hernia (*n* = 3), congenital pulmonary airway malformation (*n* = 2), esophageal atresia (*n* = 2), cloacal anomaly (*n* = 1), omphalocele (*n* = 1), congenital small‐bowel atresia (*n* = 1), hydrops fetalis (*n* = 1), and transverse facial cleft (*n* = 1).

Changes in maternal hemodynamic parameters before and after controlled‐release nifedipine administration are shown in Table 2. Systolic blood pressure significantly decreased from 110.2 ± 9.1 mmHg before administration to 107.5 ± 10.2 mmHg after administration. The mean change was −2.7 mmHg (95% CI, −4.5 to −0.8 mmHg; *p* < 0.01). Diastolic blood pressure was 65.4 ± 7.7 mmHg before administration and 64.8 ± 7.7 mmHg after administration, with no significant change observed (mean change, −0.7 mmHg; 95% CI, −2.2 to 0.8 mmHg; *p* = 0.35). Nine patients (23.7%) had at least one episode of systolic blood pressure < 90 mmHg before administration compared to 12 patients (31.6%) after administration, with no significant difference found (*p* = 0.55). None of the patients had a systolic blood pressure of < 80 mmHg. The proportion of patients with at least one episode of diastolic blood pressure < 60 or < 50 mmHg did not differ significantly before and after administration. None of the patients showed a decrease in blood pressure of ≥ 15 mmHg from baseline. Heart rate significantly decreased from 95.3 ± 14.5 beats/min before administration to 90.9 ± 12.4 beats/min after administration. The mean change was −4.4 beats/min (95% CI, −7.4 to −1.3 beats/min; *p* < 0.01). The number of patients with at least one episode of heart rate ≥ 120 beats/min decreased from 12 (31.6%) before administration to 5 (13.2%) after administration, although the difference was not statistically significant (*p* = 0.07). Changes in other maternal vital signs before and after controlled‐release nifedipine administration are shown in Table 3. Respiratory rate and SpO_2_ did not change significantly after nifedipine administration. Body temperature significantly decreased from 36.9°C ± 0.2°C before administration to 36.8°C ± 0.2°C after administration; however, the mean change was small (−0.1°C; 95% CI, −0.2°C to 0.0°C; *p* < 0.01).

**TABLE 2:** Maternal hemodynamic parameters before and after controlled‐release nifedipine administration.

	Before administration	After administration	Change from baseline	*p*
SBP (mmHg)	110.2 ± 9.1	107.5 ± 10.2	−2.7 (−4.5 to −0.8)	< 0.01
Patients with at least one SBP < 90 mmHg	9 (23.7)	12 (31.6)		0.55
Patients with at least one SBP < 80 mmHg	0 (0)	0 (0)		—
SBP reduction ≥ 15 mmHg from baseline		0 (0)		
DBP (mmHg)	65.4 ± 7.7	64.8 ± 7.7	−0.7 (−2.2 to 0.8)	0.35
Patients with at least one DBP < 60 mmHg	29 (76.3)	30 (78.9)		1.00
Patients with at least one DBP < 50 mmHg	11 (28.9)	6 (15.8)		0.13
DBP reduction ≥ 15 mmHg from baseline		0 (0)		
Heart rate (beats/min)	95.3 ± 14.5	90.9 ± 12.4	−4.4 (−7.4 to −1.3)	< 0.01
Patients with at least one heart rate ≥ 120 beats/min	12 (31.6)	5 (13.2)		0.07

*Note:* Data are presented as mean ± standard deviation, *n* (%), or mean difference with a 95% confidence interval. The change from baseline was calculated as the difference between the values after nifedipine administration and the baseline values. Categorical variables indicated the number of patients with at least one measurement meeting each criterion. *p*‐values for continuous variables were calculated using paired tests and those for categorical variables were calculated using the exact McNemar test. The *p*‐value was not calculated when no discordant pairs were observed.

Abbreviations: DBP, diastolic blood pressure; SBP, systolic blood pressure.

**TABLE 3:** Maternal vital signs before and after controlled‐release nifedipine administration.

	Before administration	After administration	Change from baseline	*p*
Respiratory rate (breaths/min)	17.4 ± 0.6	17.4 ± 0.5	0.0 (−0.1 to 0.2)	0.62
SpO_2_ (%)	97.8 ± 0.5	97.9 ± 0.4	0.1 (−0.1 to 0.2)	0.42
Body temperature (°C)	36.9 ± 0.2	36.8 ± 0.2	−0.1 (−0.2 to 0.0)	< 0.01

*Note:* Data are presented as mean ± standard deviation or mean difference with 95% confidence interval. The change from baseline was calculated as the difference between the values after nifedipine administration and the baseline values. The *p*‐values were calculated using paired *t*‐tests.

Abbreviation: SpO_2_, peripheral oxygen saturation.

To assess whether transient hemodynamic changes occurred around the expected peak plasma concentration of controlled‐release nifedipine, we performed a sub‐analysis focusing on this time window. No significant reduction in blood pressure or other clinically relevant changes in vital signs were observed (Table 4).

**TABLE 4:** Maternal hemodynamic parameters during the expected peak plasma concentration window of controlled‐release nifedipine (*n* = 28).

	Before administration	After administration	Change from baseline	*p*
SBP (mmHg)	109.3 ± 10.6	106.7 ± 10.0	−2.6 (−5.8 to 0.6)	0.11
Patients with at least one SBP < 90 mmHg	3 (10.7)	4 (14.3)		1.00
Patients with at least one SBP < 80 mmHg	0 (0)	0 (0)		—
SBP reduction ≥ 15 mmHg from baseline		0 (0)		
DBP (mmHg)	64.7 ± 8.2	63.2 ± 8.9	−1.6 (−3.9 to 0.8)	0.18
Patients with at least one DBP < 60 mmHg	15 (53.6)	16 (57.1)		1.00
Patients with at least one DBP < 50 mmHg	4 (14.3)	4 (14.3)		1.00
DBP reduction ≥ 15 mmHg from baseline		0 (0)		
Heart rate (beats/min)	93.8 ± 13.9	91.5 ± 13.2	−2.3 (−6.0 to 1.4)	0.22
Patients with at least one heart rate ≥ 120 beats/min	4 (14.3)	1 (3.6)		0.38
Respiratory rate (breaths/min)	17.5 ± 0.7	17.3 ± 0.9	−0.2 (−0.5 to 0.1)	0.20
SpO_2_ (%)	97.7 ± 0.6	97.7 ± 0.5	0.0 (−0.3 to 0.3)	0.96
Body temperature (°C)	36.9 ± 0.3	36.8 ± 0.3	−0.1 (−0.2 to 0.0)	0.08

*Note:* Data are presented as mean ± standard deviation, *n* (%), or mean difference with a 95% confidence interval. The change from baseline was calculated as the difference between the values after nifedipine administration and the baseline values. Categorical variables indicated the number of patients with at least one measurement meeting each criterion. *p*‐values for continuous variables were calculated using paired tests and those for categorical variables were calculated using the exact McNemar test. The *p*‐value was not calculated when no discordant pairs were observed.

Abbreviations: DBP, diastolic blood pressure; SBP, systolic blood pressure.

Adverse maternal effects of controlled‐release nifedipine are shown in Table 5. Headache was the most frequent adverse effect observed in 11 patients (28.9%); however, only three of these patients required analgesic medication. Palpitations and nausea were observed in two patients (5.3%), whereas facial flushing and other symptoms were observed in one patient (2.6%). During the postadministration period, controlled‐release nifedipine was temporarily withheld in two patients because of decreased blood pressure, whereas no patients required temporary withholding because of other adverse effects. In addition, none of the patients ultimately discontinued controlled‐release nifedipine due to adverse effects, including hypotension. During the postadministration period, no emergency delivery was performed directly because of hypotension or the adverse effects of nifedipine administration. A review of cardiotocography recordings and medical records revealed no emergency cesarean deliveries for nonreassuring fetal status potentially associated with maternal hypotension after nifedipine administration, and no patients required intrauterine resuscitation. The indications for cesarean delivery are summarized in Table S3, and none of the cesarean deliveries were performed due to nonreassuring fetal status.

**TABLE 5:** Maternal adverse effects and clinical events during controlled‐release nifedipine treatment.

Adverse effects of nifedipine	
Headache	11 (28.9)
Palpitations	2 (5.3)
Facial flushing	1 (2.6)
Nausea	2 (5.3)
Other symptoms	1 (2.6)
Discontinuation due to adverse effects	0 (0)
Dose omission due to hypotension	2 (5.3)
Dose omission due to adverse effects	0 (0)
Emergency delivery related to maternal hypotension	0 (0)

*Note:* Data are presented as *n* (%). The percentages were calculated using the total number of patients treated with nifedipine as the denominator.

## 4 Discussion

In this study, we evaluated maternal hemodynamic parameters, including blood pressure and heart rate, other vital signs, incidence of hypotension, and maternal and fetal adverse effects before and after the administration of controlled‐release nifedipine in 38 normotensive pregnant women with threatened preterm labor. The main finding of this study was that systolic blood pressure significantly decreased after controlled‐release nifedipine administration; however, the mean reduction was small, at −2.7 mmHg. Diastolic blood pressure also showed a slight but nonsignificant decrease, and heart rate significantly decreased by 4.4 beats/min after administration. In contrast, the incidence of hypotension did not significantly differ before and after nifedipine administration, and no serious adverse maternal effects or emergency deliveries related to maternal hypotension were observed. However, in a sub‐analysis of 28 patients with available vital sign measurements during the time window encompassing the expected peak plasma concentration, no significant changes in blood pressure, heart rate, or other vital signs were observed after controlled‐release nifedipine administration. These findings suggest that controlled‐release nifedipine is associated with modest changes in maternal hemodynamic parameters in normotensive pregnant women without evidence of clinically significant hemodynamic instability under appropriate monitoring.

When nifedipine, an antihypertensive agent, is used to treat threatened preterm labor in normotensive pregnant women, excessive blood pressure reduction and its potential effect on placental circulation are important concerns [14, 15, 16]. In the present study, the mean changes in systolic and diastolic blood pressure after controlled‐release nifedipine administration were modest. None of the patients experienced a decrease of ≥ 15 mmHg in systolic or diastolic blood pressure from baseline. Furthermore, no significant changes in the maternal hemodynamic parameters were observed during the time window encompassing the expected peak plasma concentration after controlled‐release nifedipine administration. In contrast, Luewan et al. evaluated the hemodynamic effects of immediate release nifedipine in 157 normotensive pregnant women with threatened preterm labor and reported significant decreases in systolic (4.5 mmHg; 95% CI, 2.7–6.2 mmHg) and diastolic (4.2 mmHg; 95% CI, 2.8–5.5 mmHg) blood pressure at 60 min after nifedipine administration [9]. They also reported that approximately 17% of patients had a ≥ 15 mmHg decrease in blood pressure from baseline within 60 min. Similarly, Yamasato et al. retrospectively evaluated 138 nonhypertensive pregnant women who received immediate release nifedipine for tocolysis and reported that the mean systolic and diastolic blood pressures decreased by 5 mmHg during the first 8 h after treatment initiation [10]. However, in that study, the incidence of hypotension did not increase significantly and no emergency deliveries related to hypotension or fetal heart rate abnormalities were observed.

Although Luewan et al. reported that a relatively large proportion of patients had a decrease in blood pressure of ≥ 15 mmHg, severe maternal hypotension or apparent adverse fetal effects were not observed [9]. Our findings are consistent with these previous reports in that systolic blood pressure significantly decreased after nifedipine administration; however, the magnitude of the reduction was small and no maternal or fetal adverse events related to severe hypotension were observed. Our study differs from previous studies in that it focused on a controlled‐release formulation [9, 10]. Immediate release nifedipine produces a relatively rapid increase in plasma concentration and may therefore be more likely to cause early blood pressure reduction or tachycardia. In contrast, controlled‐release nifedipine has a slower drug‐release profile and may suppress abrupt increases in plasma concentrations, potentially reducing the risk of acute blood pressure reduction. The mild reduction in blood pressure and the absence of serious adverse effects observed in this study may reflect the pharmacokinetic characteristics of the controlled‐release formulation. These findings suggest that controlled‐release nifedipine can be used for the maintenance of tocolysis, while avoiding abrupt hemodynamic changes.

Regarding heart rate, reflex tachycardia due to the vasodilatory effect of nifedipine was expected. However, in the present study, heart rate significantly decreased after nifedipine administration, and the number of patients with at least one episode of heart rate ≥ 120 beats/min also decreased after administration. This finding is unlikely to indicate a direct heart rate‐lowering effect of nifedipine. Rather, it may reflect the reduction or discontinuation of other tocolytic agents, such as intravenous ritodrine hydrochloride, improvement in uterine contractions, or improvement in maternal symptoms. In this study, controlled‐release nifedipine was used as an add‐on therapy, an alternative therapy for dose reduction of other tocolytic agents, or for supportive purposes when discontinuing intravenous tocolytic agents. Therefore, the decrease in heart rate after nifedipine administration reflects the overall clinical course and treatment strategy rather than the pharmacological effects of nifedipine alone.

Respiratory rate and SpO_2_ did not significantly change after nifedipine administration. Body temperature showed a significant decrease; however, the mean change was only −0.1°C and was therefore considered to be of limited clinical significance. Nifedipine is not generally considered to directly affect thermoregulation. Thus, the slight decrease in body temperature observed in this study may have been influenced by multiple factors including dose reduction or discontinuation of ritodrine hydrochloride, which can be associated with sweating or fever, improvements in uterine contractions, and changes in concomitant medications [17].

Headache was the most frequent maternal adverse effect. Other adverse effects, such as palpitations, nausea, and facial flushing, were infrequent. Luewan et al. reported mild adverse effects in seven of 157 patients, including palpitations in six patients (3.8%) and mild headache in one patient [9]. Headache and facial flushing are typical adverse effects associated with nifedipine's vasodilatory effects. The reported frequency of headaches varies across studies. In a retrospective study conducted in Hong Kong, moderate headaches were observed in 9 of 203 women (4.4%) [16]. When mild cases were included, Guclu et al. reported headache in 25% of the women in their Doppler study [18]. In the present study, mild headache was observed in 11 women (28.9%), whereas analgesic treatment was required in only three women (7.9%). Therefore, these findings are comparable to those of previous studies. However, direct comparisons should be made with caution because the adverse effects in the present study were collected retrospectively from medical and nursing records, the treatment duration was longer in some patients, and other tocolytic agents were used concomitantly in some cases.

The clinical significance of this study lies in providing information on the safety of controlled‐release nifedipine for treating threatened preterm labor. These findings indicate that controlled‐release nifedipine causes only a mild decrease in systolic blood pressure, with no apparent adverse effects attributable to severe hypotension. Therefore, under appropriate monitoring, including blood pressure assessment during the early phase of treatment, controlled‐release nifedipine may be a feasible option for maintenance or adjunctive therapy when other tocolytic agents are being reduced or discontinued in women with threatened preterm labor.

The strength of this study is that it focused on controlled‐release nifedipine and evaluated hemodynamic changes before and after its administration in normotensive pregnant women in Japan. In Japan, the use of nifedipine for threatened preterm labor is off‐label and safety data for pregnant women are limited. These findings provide useful real‐world clinical data for safety assessment and monitoring strategies when controlled‐release nifedipine is used off‐label in Japanese clinical settings. Furthermore, we compared the incidence of hypotension and tachycardia 1 week before and after nifedipine administration. Previous studies evaluated the frequency of hypotension and tachycardia after nifedipine administration but did not sufficiently assess how often these findings were already present before treatment [9, 10]. Therefore, it was difficult to determine whether these events occurred after nifedipine administration or reflected preexisting patient characteristics. We directly evaluated the changes associated with controlled‐release nifedipine treatment by comparing the incidences of hypotension and tachycardia before and after administration.

This study had some limitations. First, hemodynamic parameters, such as blood pressure and heart rate, may be affected by various factors, including nifedipine dose, gestational age, measurement position, uterine contractions, infection, and concomitant medications; however, it was difficult to fully adjust for these potential confounders. Previous studies, including those using animal models, have reported decreased and increased maternal blood pressure associated with ritodrine hydrochloride; however, no consistent conclusion has been reached regarding its hemodynamic effects [19, 20]. In contrast, magnesium sulfate is associated with a decrease in mean arterial pressure [21]. Therefore, dose changes or discontinuation of these agents may have influenced the hemodynamic parameters, including blood pressure and heart rate, making it difficult to determine whether the changes observed in this study were attributable to nifedipine alone. Second, this was a retrospective observational study, and the timing and frequency of blood pressure and heart rate measurements were not fully standardized. Therefore, transient blood pressure reduction or tachycardia shortly after administration may not have been fully captured. Third, adverse effects were not prospectively and comprehensively assessed but were collected from physician and nursing records; thus, mild symptoms may have been underreported. Additionally, many patients received concomitant ritodrine hydrochloride or magnesium sulfate, and the observed adverse effects could not be definitively attributed to nifedipine alone. Fourth, the sample size was limited to 38 patients, and the study was underpowered to detect rare but serious adverse events.

In conclusion, controlled‐release nifedipine in normotensive pregnant women was associated with a small decrease in systolic blood pressure without serious maternal and fetal adverse events attributable to maternal hypotension. Heart rate decreased after administration, whereas respiratory rate and SpO_2_ showed no apparent changes. Although headaches were relatively common, none of the patients discontinued treatment because of adverse effects. These findings suggest that controlled‐release nifedipine may be a feasible option for maintenance or adjunctive tocolytic therapy in normotensive pregnant women when used with appropriate maternal monitoring.

## Author Contributions

**Takafumi Ushida:** conceptualization, data curation, formal analysis, writing – original draft, investigation, funding acquisition, methodology. **Sumire Fujito:** conceptualization, formal analysis, investigation, writing – review and editing. **Yoshitaka Shiraishi:** conceptualization, formal analysis, investigation, writing – review and editing. **Yuki Nozaki:** conceptualization, formal analysis, investigation, writing – review and editing. **Kazuya Fuma:** conceptualization, formal analysis, investigation, writing – review and editing, supervision. **Sho Tano:** conceptualization, formal analysis, investigation, supervision, writing – review and editing. **Seiko Matsuo:** conceptualization, formal analysis, investigation, supervision, writing – review and editing. **Hiroaki Kajiyama:** conceptualization, formal analysis, supervision, investigation, writing – review and editing.

## Funding

This study was supported by a grant from the Japan Society for the Promotion of Science (JSPS KAKENHI; 25K14888), awarded to T.U.

## Disclosure

The authors have nothing to report.

## Ethics Statement

The study protocol was approved by the Institutional Review Board of Nagoya University Hospital on March 4, 2016 (approval No. 2015‐0415). The present study was approved by subsequent protocol amendments, with the final approval granted on June 30, 2026. Because this was a retrospective study that used information from medical records, the requirement for individual informed consent for study participation was addressed using an opt‐out approach.

## Consent

Because this was a retrospective observational study using information obtained from medical records, the requirement for individual informed consent for study participation was addressed using an opt‐out approach approved by the Institutional Review Board of Nagoya University Hospital. Separately, because nifedipine is used off‐label for threatened preterm labor in Japan, the expected benefits and potential adverse effects of off‐label nifedipine use were explained to all patients, and written informed consent for nifedipine treatment was obtained before administration.

## Conflicts of Interest

Hiroaki Kajiyama is the editor‐in‐chief of the Journal of Obstetrics and Gynecology Research and co‐author of this article. He should be excluded from editorial decision making related to the acceptance and publication of this article. An Associate Editor should independently handle editorial decision‐making to remove conflicts of interest and minimize bias. The other authors declare no conflicts of interest.

## Supporting information


**Table S1:** Pharmacokinetic parameters of different nifedipine formulations after a single oral dose.
**Table S2:** Number of vital sign measurements before and after controlled‐release nifedipine administration.
**Table S3:** Indications for cesarean delivery.

## Data Availability

The individual‐level data used in this study are not publicly available because they contain clinical information obtained from the medical records of pregnant women and their infants, and public sharing of these data was not included in the ethical approval or the opt‐out information provided to the study participants. Therefore, depositing the individual‐level data in a public repository would not comply with the conditions under which the study was approved by the Institutional Review Board of Nagoya University Hospital. Researchers who wish to access the data for scientifically appropriate purposes may contact the corresponding author. Requests will be considered on a case‐by‐case basis and will require approval by the Institutional Review Board of Nagoya University Hospital and compliance with applicable institutional and ethical requirements.
