# Supplementary material for: Hemodynamic Effects of Controlled‐Release Nifedipine for Tocolysis in Normotensive Pregnant Women
Source: J Obstet Gynaecol Res. 2026 Nov 10;52(11):e70560. doi: 10.1111/jog.70560 (PMC13749575; doi:10.1111/jog.70560)
Supplement: Supplementary file 1 [file JOG-52-0-s001.docx]

Supplementary Table 1. Pharmacokinetic parameters of different nifedipine formulations after a single oral dose

| Formulation | Product | Dose | Cmax (ng/mL) | Tmax (h) | T1/2 (h) | AUC |
| --- | --- | --- | --- | --- | --- | --- |
| Immediate release | Nifedipine capsule | 10 mg | 163 ± 44 | 0.5 ± 0.1 | 3.0 ± 1.4 | 245 ± 37 ng·h/mL (0–8 h) |
| Sustained release | Nifedipine L tablet | 20 mg | 75.0 ± 17.3 | 2.8 ± 0.4 | 3.9 ± 1.7 | 435.5 ± 112.2 ng·h/mL (0–36 h) |
| Controlled-release | Nifedipine CR tablet | 20 mg | 26.2 ± 12.4 | 3.1 ± 2.6 | NA | 315.2 ± 124.8 ng·h/mL (0–48 h) |

Data are presented as mean ± standard deviation. The pharmacokinetic parameters were obtained after a single oral administration of each nifedipine formulation under fasting conditions in healthy adult men. Cmax, maximum plasma concentration; Tmax, time to maximum plasma concentration; T1/2, elimination half-life; AUC, area under the plasma concentration–time curve; NA, not available; CR, controlled release.

Supplementary Table 2. Number of vital signs measured before and after controlled-release nifedipine administration

|  | Before administration | | After administration | |
| --- | --- | --- | --- | --- |
|  | Measurements per patient, median (IQR) | Total measurements | Measurements per patient, median (IQR) | Total measurements |
| Systolic blood pressure | 15.5 (11.3 to 21) | 597 | 17 (14 to 20) | 606 |
| Diastolic blood pressure | 15.5 (11.3 to 21) | 597 | 17 (14 to 20) | 606 |
| Heart rate | 15.5 (12 to 21) | 596 | 16.5 (13.3 to 20) | 599 |
| Respiratory rate | 15 (11 to 21) | 586 | 14 (12 to 17) | 518 |
| SpO₂ | 15 (11 to 21) | 583 | 14 (12 to 17.8) | 516 |
| Body temperature | 15 (11 to 21) | 576 | 15 (12 to 19) | 512 |

Data are presented as the median number of measurements per patient, with an interquartile range, and the total number of measurements during each observation period. IQR, interquartile range; SpO₂, peripheral oxygen saturation.

Supplementary Table 3. Indications for cesarean delivery

| Indication for cesarean delivery | Number of cases |
| --- | --- |
| Severe fetal anomalies | 5 |
| Severe fetal growth restriction | 3 |
| Placenta previa | 3 |
| Low-lying placenta | 1 |
| Vasa previa | 1 |
| Breech presentation | 2 |
| History of uterine surgery | 3 |
| Preterm prelabor rupture of membranes at 25 weeks of gestation | 1 |

Data are presented as the number of cases for each indication.
